# Response of eelgrass (*Zostera marina*) to an adjacent Olympia oyster restoration project

**DOI:** 10.1371/journal.pone.0258119

**Published:** 2021-10-07

**Authors:** Sara Briley, Rick Ware, Christine Whitcraft, Danielle Zacherl

**Affiliations:** 1 Department of Biological Science, California State University Fullerton, Fullerton, California, United States of America; 2 Coastal Resources Management, Laguna Niguel, California, United States of America; 3 Department of Biological Sciences, California State University Long Beach, Long Beach, California, United States of America; Bigelow Laboratory for Ocean Sciences, UNITED STATES

## Abstract

Recent restoration efforts for the native Olympia oyster, *Ostrea lurida*, are commonly motivated by potential return of oyster-associated ecosystem services, including increased water filtration. The potential impact of such restoration on another species of ecological concern, eelgrass, *Zostera marina*, is unclear, but has been hypothesized to be positive if oyster filter feeding increases light penetration to eelgrass. For two years after construction of an oyster restoration project, we assessed the response of adjacent eelgrass (impact) compared to control and reference eelgrass beds by monitoring changes in light intensity, eelgrass shoot density, biomass, leaf morphometrics, and epiphyte load. We observed lower light intensity consistently over time, including prior to restoration, near the constructed oyster bed relative to the control and one of the reference locations. We also observed minor variations between control and impact eelgrass morphology and density. However, the changes observed were not outside the range of natural variation expected in this system, based upon comparisons to reference eelgrass beds, nor were they detrimental. This limited impact to eelgrass may be due in part to the incorporation of a buffer distance between the restored oyster bed and the existing eelgrass bed, which may have dampened both positive and negative impacts. These findings provide evidence that Olympia oyster restoration and eelgrass conservation goals can be compatible and occur simultaneously.

## Introduction

Large declines in historic Olympia oyster, *Ostrea lurida*, populations to the point of functional extinction [1] have promoted recent interest in oyster restoration along the west coast of the United States. In addition to increasing local abundances of the Olympia oyster, restoration practitioners are motivated by the potential recovery of ecosystem services that the oyster may provide. The eastern oyster, *Crassostrea virginica*, native to the east coast of the United States, can increase habitat complexity and community diversity, improve water clarity, cycle nutrients, and stabilize sediments [2–5]. The ecosystem services that *O*. *lurida* provides have only rarely been evaluated [e.g., 6–9] but are assumed to be similar to those provided by other oyster species, such as the eastern oyster.

In California, *Ostrea lurida* habitat has been associated with the dominant seagrass species, eelgrass, *Zostera marina*, historically and more recently. Pleistocene fossil deposits from northern California containing both species [10] support the historical association between eelgrass and the Olympia oyster, and Olympia oysters in small abundances were found within eelgrass beds in San Diego Bay, CA [11]. Elsewhere in southern California, we have observed some evidence of overlap in *O*. *lurida* and *Z*. *marina* distributions, but more commonly, *O*. *lurida* are found at a relatively higher tidal elevation [12], with *Z*. *marina* occurring relatively lower in the intertidal and extending to the shallow subtidal zone [13]. Since both species can inhabit the lower intertidal zone, there is a high potential for Olympia oysters to live within or adjacent to eelgrass beds. As Olympia oyster restoration efforts increase in number and size, the potential for these two species to interact also increases.

Bivalves and seagrass can interact and influence each other through a variety of mechanisms, though the overall direction and magnitude of impacts are inconsistent and case-specific [14, 15]. Although bivalve-mediated improvements to water or sediment conditions are expected to facilitate seagrass, in part by improving light availability upon which seagrass growth and abundance rely, this effect is highly context-dependent, depending on existing stressors, spatial configuration, and bivalve density and type [14, 15]. This facilitative effect via improvements to water clarity has been evaluated for other oyster species on seagrass [16, 17], though this impact by *O*. *lurida* on *Z*. *marina* is not well understood.

Seagrass species have some of the largest light requirements of all plants, with some species requiring up to 25% incident radiation, compared to about 1% for most angiosperms [18], which makes seagrass productivity closely tied to water clarity. Despite this requirement, seagrass exhibit well-documented physiological, morphological, and meadow-scale responses to acclimate to suboptimal light conditions [reviewed in 19, 20]. To optimize photosynthesis in light-limited conditions, seagrasses first undergo physiological alterations at the cellular level and then, if the conditions continue or worsen, progress to alterations observable at the plant-scale, such as reduced growth and altered leaf size, and then to the meadow-scale, such as declines in shoot density and biomass [21–24]. The morphological plasticity observed in response to changes in light availability allows seagrass populations to persist in suboptimal conditions and also makes monitoring these parameters of seagrass useful bioindicators of light stress [20].

Oysters may improve water clarity both through their filtration activity and via their creation of additional three-dimensional structure. By removing phytoplankton and suspended sediments out of the water column through filter feeding, oysters may increase light available to eelgrass blades for photosynthesis [reviewed in 25] which can translate into increased seagrass growth [26, 27]. The complex structure of oyster beds as an aggregate of both adult oysters and vacated oyster shell can also improve water clarity for eelgrass growth through wave attenuation and physical stabilization of the sediment [2, 16, 28, 29]. The structure of an oyster bed could thus prevent fine particles that would decrease light penetration to eelgrass from becoming re-suspended in the water.

Oysters may additionally improve light penetration to eelgrass by altering the light reaching the eelgrass leaf surface through reductions in epiphytic load. Epiphytes, organisms that grow upon or are attached to the eelgrass blades, act as an additional barrier to eelgrass in attaining light requirements for photosynthesis, as less surface area of the leaf blade is exposed to light [30]. Oysters may decrease eelgrass epiphytic loads by increasing the amount of habitat complexity available as predation refuge to epiphyte grazers [31–33]. The presence of additional epiphyte grazers may decrease the coverage of epiphytic organisms and allow more light penetration for increased eelgrass growth [34].

Despite evidence of the potential benefits of oysters for eelgrass, there is also evidence of negative impacts associated with higher densities of oysters. Increased oyster densities have led to a decline in eelgrass cover, plant size, biomass, and growth, likely due to space competition as well as build-up of toxic sulfide levels from enriched oyster bio-deposits [33, 35–38].

It is unclear whether the Olympia oyster could have the same impact on eelgrass as previously studied oyster species due to its much smaller size, bed structure, and lower water filtering capabilities compared to the larger oyster species more commonly studied [8, 39]. In addition, prior studies were almost exclusively done by placing oysters directly within eelgrass beds [e.g., 33, 36], which does not accurately reflect the observed zonation of the two species, at least in southern California, or the arrangement typically used in oyster restoration projects. It is unclear whether the benefits remain, and negative impacts are diminished when native Olympia oysters are placed adjacent to, rather than within, existing eelgrass beds.

Seagrass is also a target of restoration and conservation focus along the west coast of the United States due to substantial population declines and provision of many critical ecosystem services [40]. Seagrasses provide many similar ecosystem services as oyster beds, including habitat for associated species [41], nursery grounds [42] and sediment stabilization [16, 43]. In addition, seagrass is also a major primary producer [44, 45] and a large contributor of carbon to detrital pathways [46]. Seagrass canopies can increase sedimentation of suspended particles and improve water clarity by altering water flow through the resistance of the blades [43]. The conservation and protection of valuable eelgrass habitat is a priority for natural resource managers; eelgrass is protected by state and federal law under the Clean Water Act, the Magnuson-Stevens Fishery Conservation and Management Act, and the California Code of Regulations. According to these laws and regulations, activities that impact eelgrass habitat and potential habitat should be first avoided, then minimized, and if unavoidable, mitigated. As such, it is important for future oyster restoration efforts to avoid damage to existing eelgrass beds, and to document any benefits and/or disadvantages of restoring the two species in close association.

An Olympia oyster restoration project constructed shoreward of eelgrass habitat offered an opportunity to clarify the impact of Olympia oyster restoration on eelgrass. By observing eelgrass at locations with and without an adjacent constructed oyster bed, both before and after construction, we addressed the following research questions: (a) Does the presence of the constructed oyster bed increase light available for eelgrass over time? (b) Does the presence of the constructed oyster bed affect eelgrass shoot density, biomass (above- or belowground), or leaf morphology (length or width) over time? (c) Does the presence of the constructed oyster bed lead to a decrease in epiphytes (biomass or percent cover) on eelgrass leaves over time?

## Materials and methods

### Ethics statement

All necessary permits were obtained for the described study. Multiple permits and certifications were required to construct the oyster bed, which included a Coastal Development Permit Waiver from the California Coastal Commission (E-11-006-W), a Nationwide Permit No. 27 for Aquatic Habitat Restoration, Establishment, and Enhancement Activities from the Army Corps of Engineers (SPL-2011-00381-JWM), a General Certification and Notification to Proceed from the California State Water Resources Control Board, and a Right of Entry Permit from the City of Long Beach to enter and work at the Jack Dunster Marine Reserve. The collection of seagrass samples undertaken by this research was approved through the issuing of scientific collecting permits by the California Department of Fish and Wildlife for the duration of the study. No endangered or protected species were collected during this project.

### Study site and species

This study took place in Alamitos Bay, a highly urbanized and developed bay in the city of Long Beach, in Los Angeles County, California, with a surface area of approximately 1.2 km^2^ (285 acres) [47] and a tidal prism of approximately 1.96 x 10^6^ m^3^ [48]. The physical conditions in the bay during the time of the study were partly driven by the once-through cooling technology of the nearby AES Alamitos Generating Station and Haynes Generating Station. Cooling water uptake by the power plants in the upper portion of Alamitos Bay created a net transport of ocean water into the bay and a mean water residence time of approximately one day [48]. This high flushing rate may have improved water quality in the bay. Because of minimal seasonal freshwater inputs, Alamitos Bay is effectively a marine environment, with salinities ranging from 30–35 PSU [47, 48]. Temperatures can range from 13° C in the winter and up to 25° C in the summer [49].

*Zostera marina* beds grow on sand and mud substrata throughout the bay [50]. Both intertidal and subtidal populations are present at elevations between approximately 0 m and -2.6 m MLLW [50]. *Ostrea lurida* beds were documented in Alamitos Bay in the early 1900’s [51, 52], but current densities of the oyster are extremely low throughout the bay ranging from ~ 1.6 to 21.2 oysters m^-2^ [12]. The native oyster occurs on hard substrata in the lower intertidal to shallow subtidal, with historic extreme limits for the species observed at + 2 m above and -10 m MLLW [53], although the current lower depth limit is unclear.

### Oyster habitat restoration details

**Fig 1:**
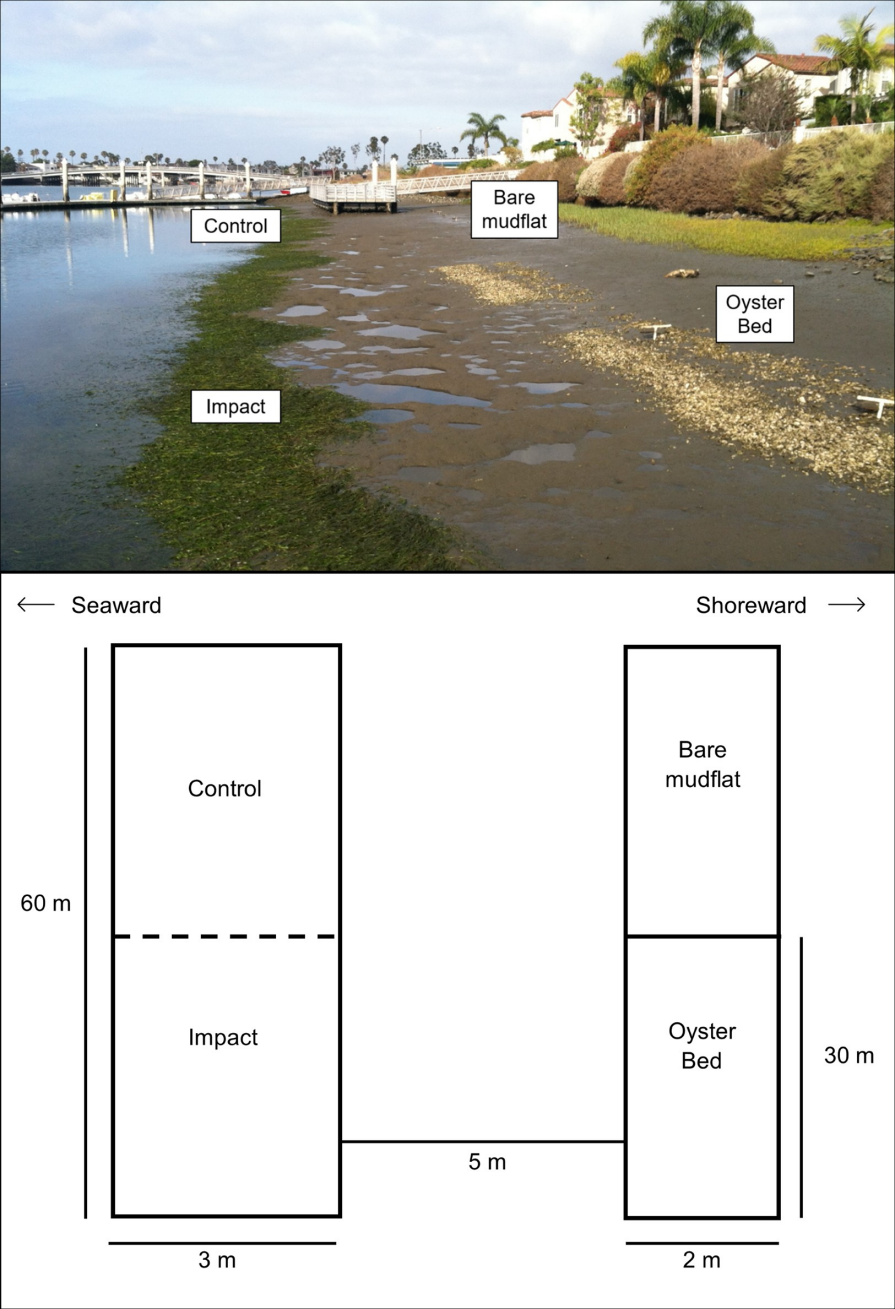
Orientation of control and impact eelgrass locations within Jack Dunster Marine Reserve (JDMR). Impact and control eelgrass locations in relation to a shoreward oyster bed constructed in June 2012.

**Fig 2:**
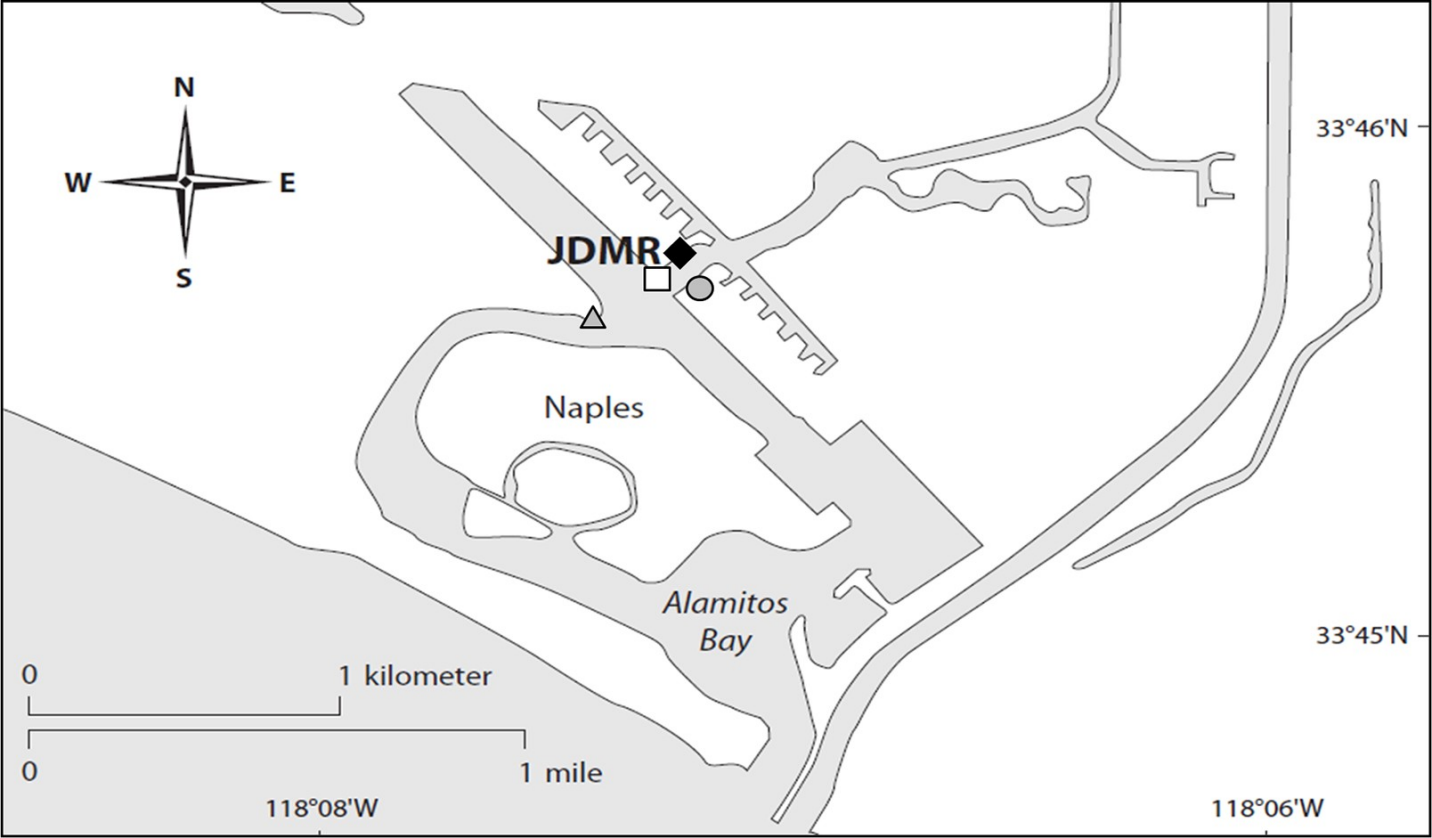
Site locations in Alamitos Bay, Long Beach, California, USA. Impact (◆) and control location (□) within Jack Dunster Marine Reserve (JDMR) in relation to reference eelgrass beds 1 (grey circle) and 2 (grey triangle).

To return missing oyster bed habitat back to Alamitos Bay, a collaborative group of scientists and non-profit organizers from California State University, Fullerton, California State University Long Beach, KZO Education, and Orange County Coastkeeper initiated an Olympia oyster restoration project in Jack Dunster Marine Reserve (JDMR) (33°45’43.98"N, 118° 7’10.74"W, Figs 1 and 2) in June 2012. Because of a lack of suitable oyster habitat, restoration involved supplementing clean empty Pacific oyster shell to the mudflat to increase the natural recruitment of oyster larvae. Shells were originally added in one long rectangular area parallel to shore (30 m by 2 m) up to 0.12 m thick at an elevation of approximately + 0.37 m MLLW. Within six months of construction, however, the oyster shell bed experienced significant shell loss (28% remaining of original cover), most likely a result of sediment deposition [54]. However, oyster larval settlement occurred, and adults were present at a density comparable to the highest density found elsewhere in the bay within the first year (June 2013: 27.43 ± 15.50 individuals m^-2^) [54]. To ameliorate shell loss, in late June 2013 more shell was added to the mudflat at a lower intertidal height (+ 0.1 to 0.2 m MLLW) and in three sections to prevent sediment deposition onto the shell bed by creating water channels. This adaptive management strategy maintained high shell percent cover through the end of the study and supported much higher densities of oysters (~ 400 oysters m^-2^) by June 2014 [54].

### Experimental design

Prior to oyster restoration, a half-acre of eelgrass habitat was present within Jack Dunster Marine Reserve (JDMR). The initial oyster bed was placed approximately 5 meters (m) inshore of the upper edge of eelgrass habitat, although this distance decreased over time as eelgrass habitat expanded inshore towards the oyster bed. To assess the impact of oyster restoration on eelgrass, we monitored two areas of eelgrass habitat before and throughout two years after construction. A 30 m by 3 m portion of the eelgrass directly seaward of the constructed oyster bed was designated as the impact location, where the greatest impact was expected, and an adjacent 30 m by 3 m portion of eelgrass seaward of an un-manipulated mudflat was designated as the control location (Fig 1). Monitoring this control location facilitated the best available comparison between eelgrass with and without a shoreward oyster bed. The lack of replication of both treatment and control plots is a common issue in the evaluation of restoration projects, as well as in environmental impact studies, though there are designs which may help alleviate this issue [reviewed in 55]. Underwood [56–58] described a design incorporating multiple control sites to compare with the impact site. Similarly, we monitored two nearby eelgrass beds of the same size and tidal height outside of JDMR to account for reference eelgrass conditions not impacted by oyster restoration (Fig 2). The first reference eelgrass bed (reference 1) was a large eelgrass bed present shoreward of a nearby residential boat dock and the second reference eelgrass bed (reference 2) was a similarly sized eelgrass bed unprotected from currents and boat wakes, unlike the other eelgrass beds.

### Constructed oyster bed impact on light

We measured underwater light illuminance using HOBO Pendant® Temperature and Light Data Loggers at each eelgrass bed shortly before and intermittently throughout two years after construction of the oyster bed. Light meters attached to the tops of floatable buoys were suspended at a fixed height above the eelgrass canopy at a depth of -0.3 m MLLW. We placed two replicate meters on the seaward edge outside of each surveyed eelgrass area approximately 0.5 m apart. Light meters were deployed for 2–3 months at a time to capture seasonal variation throughout the first year following oyster bed construction and once again in the summer two years after restoration. Due to high sedimentation rates onto the sensors, light meters were cleaned using a soft brush and only the dates immediately following cleaning were used in analysis. The HOBO loggers collected light measurements every 5 minutes. We used the maximum light value between the 2 replicate light meters at each time period in analysis to eliminate instances of shading on one of the loggers, and then calculated the mean daily light value for each location and day by averaging the maximum light values between the hours of 10:00 and 14:00, when the sun was most directly overhead. Since the HOBO loggers do not measure all of the wavelengths used by the plant in photosynthesis (photosynthetically active radiation, PAR), nor are they equally sensitive to all wavelengths, data retrieved from the HOBO loggers allow relative comparisons between locations rather than indicating the total amount of light energy available for photosynthesis. However, prior studies found that light illuminance values collected with HOBO Loggers and PAR are highly correlated [27, 59], such that high light illuminance values would suggest high PAR values.

### Eelgrass response to the constructed oyster bed

To measure shoot density, SCUBA divers counted the number of shoots within twenty 0.125 m^2^ quadrats placed at predetermined random locations within each eelgrass bed. We monitored eelgrass shoot density in each eelgrass bed before oyster bed construction (June 2012) and approximately every 3 months after for 2 years (through June 2014).

SCUBA divers collected eelgrass above-ground and below-ground samples (n = 5 bed^-1^) before initiation of the restoration project (June 2012) and each year after for two years (June 2013, June 2014). Eelgrass above- and below-ground biomass, leaf morphometrics, and eelgrass epiphytes were measured on each of these samples. Above-ground samples were collected by cutting all eelgrass shoots originating within a randomly placed 15.24 cm diameter circular frame. To determine eelgrass above-ground biomass per shoot (shoot biomass), eelgrass leaves were dried in a lyophilizer and epiphytes were gently scraped each leaf using a stiff paintbrush. The dominance of calcareous algal and bryozoan epiphytes, which were difficult to remove using other physical methods, necessitated this removal technique as described by Penhale [60]. Cleaned above-ground samples and separate epiphyte samples were then transferred to a 60°C drying oven until constant mass was achieved and recorded for each. Shoot biomass was determined by standardizing to the number of shoots per sample and averaged across all samples.

Beneath each above-ground sample, SCUBA divers collected below-ground samples using a PVC core of the same diameter (15.24 cm) to a depth of 21 cm. In the laboratory, samples were rinsed with deionized water over a 0.5 mm sieve to remove sediment and non-root or rhizome material. Remaining below-ground biomass was dried to a constant mass (to the nearest 0.01 g) at 60°C.

In the laboratory, we measured the leaf length and leaf width from all shoots in above-ground samples. Leaf length of both broken and entire leaves was measured from the base (ligule) to the tip of each leaf to the nearest mm. Leaf width was measured to the nearest 0.05 mm at half the total length of each leaf. We determined the mean leaf length per sample by averaging the length of the longest leaf per shoot (maximum leaf length). We determined the mean leaf width per sample by averaging the width of all leaves per shoot.

### Eelgrass epiphyte response to constructed oyster bed

Prior to removing epiphytes from the above-ground eelgrass samples, we measured epiphyte percent cover on the oldest portions of as many as three shoots per sample. From the two most external leaves in each shoot we cut 8 cm from the distal end of each leaf. We overlaid a transparent rectangular grid (1 mm by 5 mm) on each 8 cm leaf portion and determined the presence/absence of epiphytes at each point-intercept using a dissecting microscope. The front and back of each 8 cm portion were combined into a single percent cover value for that leaf and averaged over the two leaves of each shoot. For each sample, we calculated the average percent cover over all the shoots within the sample. We determined epiphyte biomass as the material removed from the lyophilized above-ground material, as described above in eelgrass above-ground biomass methods. Epiphyte biomass was normalized to the total above-ground eelgrass biomass, referred to hereafter as epiphyte load.

### Statistical analyses

Mean daily light intensities were compared among locations (oyster bed present (impact) and absent (control, reference 1, reference 2)) over time, including prior to restoration, using multiple paired Wilcoxon signed-rank tests to determine specific differences among locations [61]. To control for increasing risk of type I error with multiple tests, p-values were adjusted using the Bonferroni multiple testing correction method. Analyses on light data were performed using R statistical software and associated packages, version 3.5.2 [62].

For the eelgrass density survey, we analyzed the impact of the oyster bed on shoot density over time using a 2-way ANOVA for the effects of Location (control, impact, reference 1, reference 2) and Time (9 survey times over 2 years). We considered a significant interaction between Location and Time to signal a potential effect of the oyster bed, which was further explored using contrasts of interest. These included comparisons of the control and reference locations versus the impact location during each survey time, as well as contrasts between pre-restoration and each post-restoration survey time within each location. To control for increasing risk of type I error with multiple tests, Bonferroni-corrected significance thresholds were implemented, and tests were considered significant when p < 0.00085. Data were examined visually and checked for assumptions of normality and homogeneity of variance using the Shapiro-Wilk and Bartlett’s tests. Although the assumptions were not met and could not be improved through transformation, we proceeded with a parametric ANOVA because of its robustness to minor deviations from normality and moderate heterogeneity of variances when sample sizes are equal [63]. Analyses on shoot density data were performed using R statistical software and associated packages, version 3.5.2 [62].

For the eelgrass samples, the impact of oyster restoration on eelgrass biomass, leaf morphometrics and epiphyte levels was evaluated using multivariate analysis because multiple measurements were made on the same samples and these measurements are likely correlated. Due to population declines in both reference beds, sampling efforts at these locations often returned no eelgrass to measure, resulting in no data available for leaf morphometric and epiphyte variables. Because of this, the reference locations were excluded from multivariate analysis. Prior to analysis, homogeneity of variance was assessed visually for each variable. Epiphyte load data were log-transformed to meet assumptions for homogeneity of variance and all variables were normalized to make measurements of different types and scales comparable. We used non-metric, multi-dimensional scaling (nMDS) to visually examine differences between locations and years based on the characteristics measured from collected samples (shoot biomass, below-ground biomass, leaf length, leaf width, epiphyte load and epiphyte percent cover). We then tested the effect of the constructed oyster bed on eelgrass characteristics with a 2-factor permutational multifactorial analysis of variance (PERMANOVA) test, using 9999 permutations and a Euclidian distance measure. Correlation among response variables also drove the decision to use a permutational MANOVA, which is less sensitive to correlation, rather than a parametric MANOVA. Location (control, impact) and Year (2012, 2013, 2014) were included as fixed factors in the PERMANOVA.

Following the PERMANOVA, univariate permutational ANOVAs were used to test the effect of location and year and their interactions on each response variable as suggested by Quinn & Keough [64]. To account for the fact that multiple ANOVAs had been conducted, which increases the risk of type I error, the Bonferroni method was used and each ANOVA was tested at the 0.0083 significance level. Following the discovery of a significant Year × Location interaction, pairwise comparison tests were performed within each level of Year across levels of Location and vice versa. The Benjamini–Hochberg method was used to control type I error inflation by maintaining a false discovery rate of 5% for multiple tests within each variable [65, 66]. PERMANOVA and nMDS analyses were performed using Primer 6 v.6.1.11 and PERMANOVA + v.1.0.1 software package (PRIMER-E Ltd) [67].

## Results

### Constructed oyster bed impact on light

**Fig 3:**
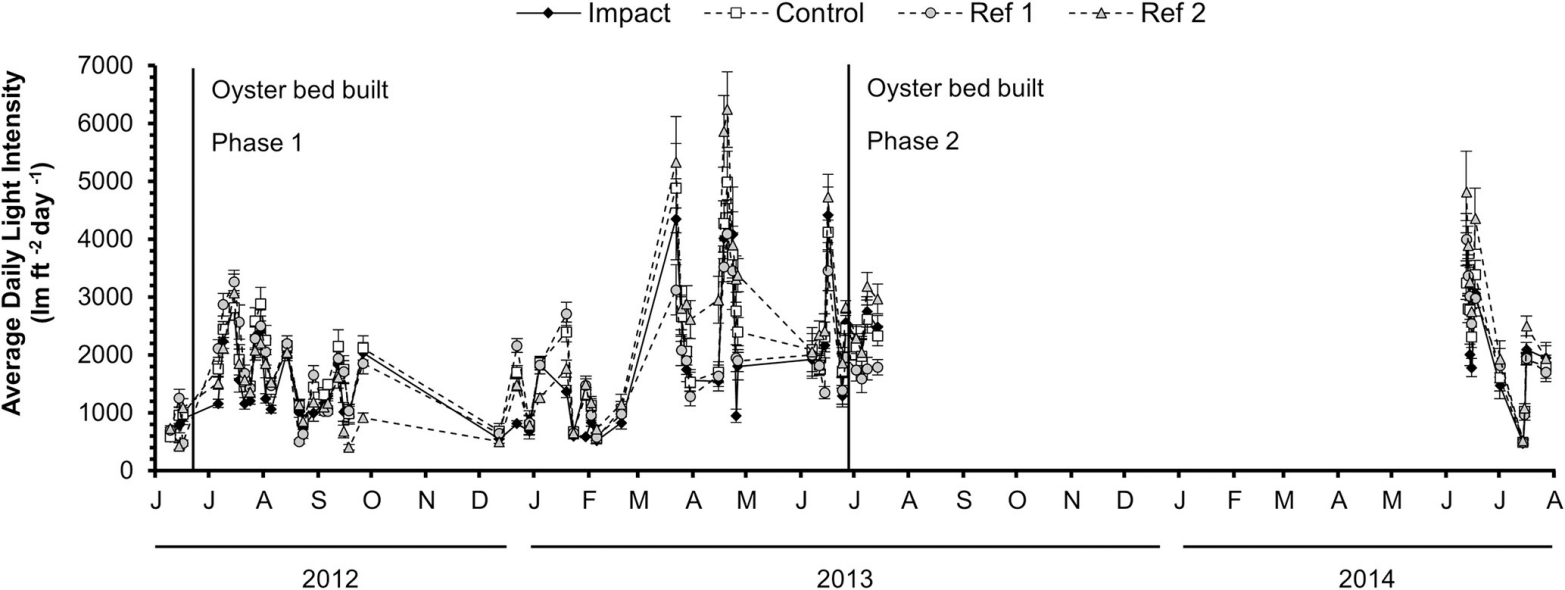
Light intensity over time. Mean daily light intensity (lumens ft^-2^ day^-1^) at each location (impact, control, reference 1, reference 2) over time. Vertical lines show timing of oyster bed constructions. Error bars show 95% confidence intervals.

Mean daily light intensity was lower at the impact location (1786.8 lm ft^-2^ day^-1^) than at the control location (2012.6 lm ft^-2^ day^-1^; Wilcoxon signed-rank test, V = 357, *p* < 0.0001) and the reference 2 location (2162.2 lm ft^-2^ day^-1^; Wilcoxon signed-rank test, V = 340.5, *p* < 0.0001), but did not differ significantly from the reference 1 location (1862.1 lm ft^-2^ day^-1^; Wilcoxon signed-rank test, V = 853, *p* = 1, Fig 3) across time. The control, reference 1, and reference 2 locations did not significantly differ from each other (Wilcoxon signed-rank tests, *p* > 0.05).

### Eelgrass response to constructed oyster bed

#### Shoot density

**Fig 4:**
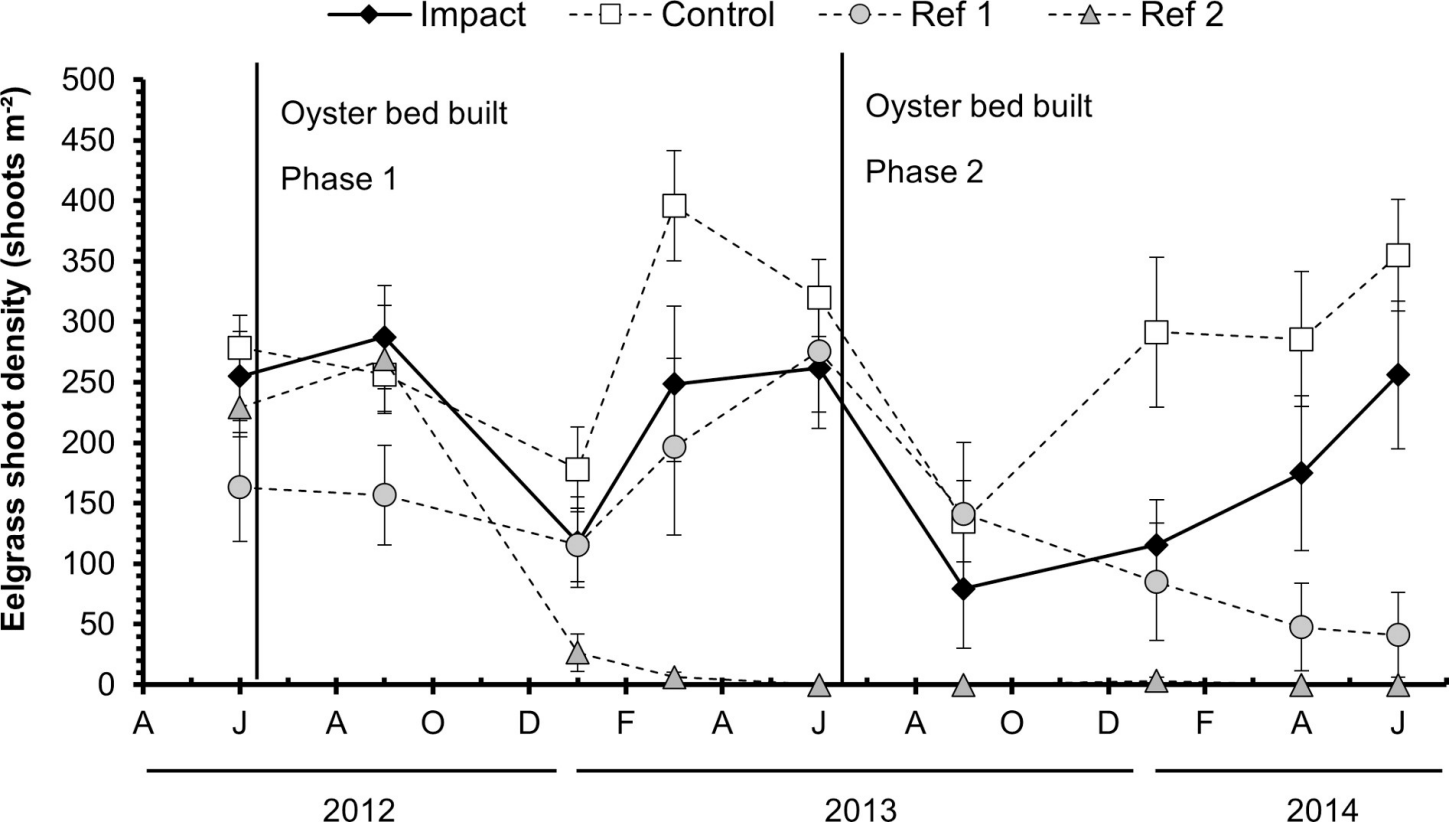
Eelgrass shoot density over time. Mean eelgrass shoot density at each location each month (n = 20 quadrats month^-1^ location^-1^). Vertical lines show timing of oyster bed constructions. Error bars show 95% confidence intervals. The results of pairwise comparisons between shoot density means are reported in S1 and S2 Tables.

Eelgrass shoot density varied among locations, but not consistently over time (2-way ANOVA, Time x Location: F_24,683_ = 12.15, *p* < 0.0001; Table 1, Fig 4). The control and impact locations did not significantly differ from each other before restoration in June 2012 or by the end of the study in June 2014, but shoot density was significantly higher in the control location than in the impact twice during the study (March 2013 and January 2014; Fig 4, S1 Table). However, shoot density at the impact location was similar to or greater than each of the reference locations at all time points (Fig 4, S1 Table).

**Table 1:** Two-way ANOVA results on eelgrass shoot density.

Factor	df	SS	F	*p* ^a^
Time	8	2156714	29.86	**< 0.0001**
Location	3	4633766	171.05	**< 0.0001**
Time × Location	24	2633424	12.15	**< 0.0001**
Residuals	683	6167536		

^a^ *p* values < 0.05 shown in bold.

Although shoot density varied over time, neither the impact nor the control locations differed from their respective pre-restoration values at the end of study in June 2014 (Fig 4, S2 Table). The reference sites ended the study with lower densities than before restoration, with declines occurring within 7 months at reference 2, but not until 22 months at reference 1 (Fig 4, S2 Table).

#### Eelgrass biomass, leaf morphometrics and epiphytes

**Fig 5:**
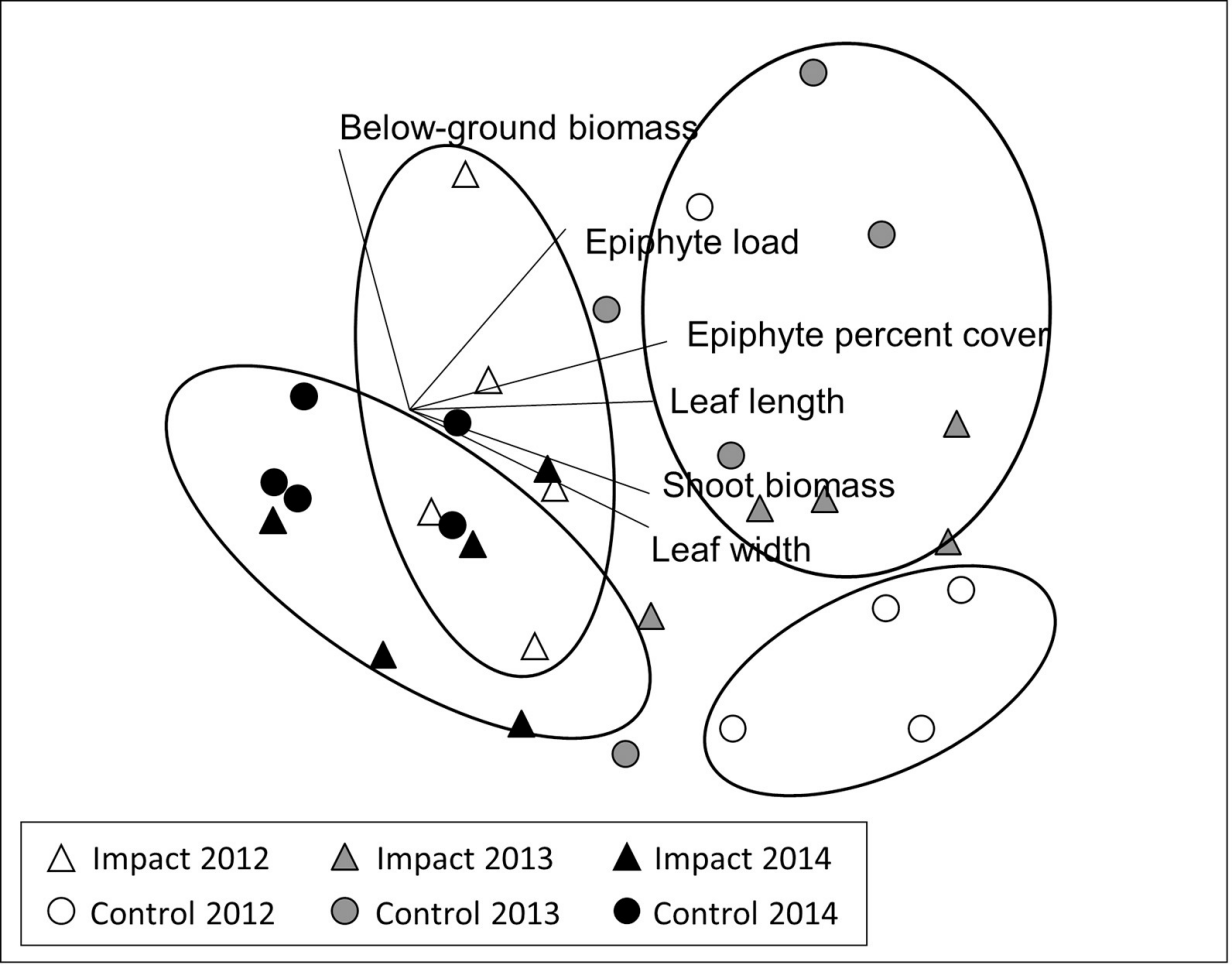
Non-metric multidimensional scaling plot illustrating variation in eelgrass characteristics between locations over time. Non-metric multidimensional scaling plot constructed from a similarity matrix based on Euclidean distance of the transformed data between the control (circle) and impact (triangle) locations across years: 2012 (white), 2013 (gray) and 2014 (black). Vectors represent Pearson correlations between variables and ordination axes, with the length and direction corresponding to the strength and sign of the correlation. Circles are drawn to indicate the pairwise test results (S3 and S4 Tables). 2D Stress: 0.14.

**Fig 6:**
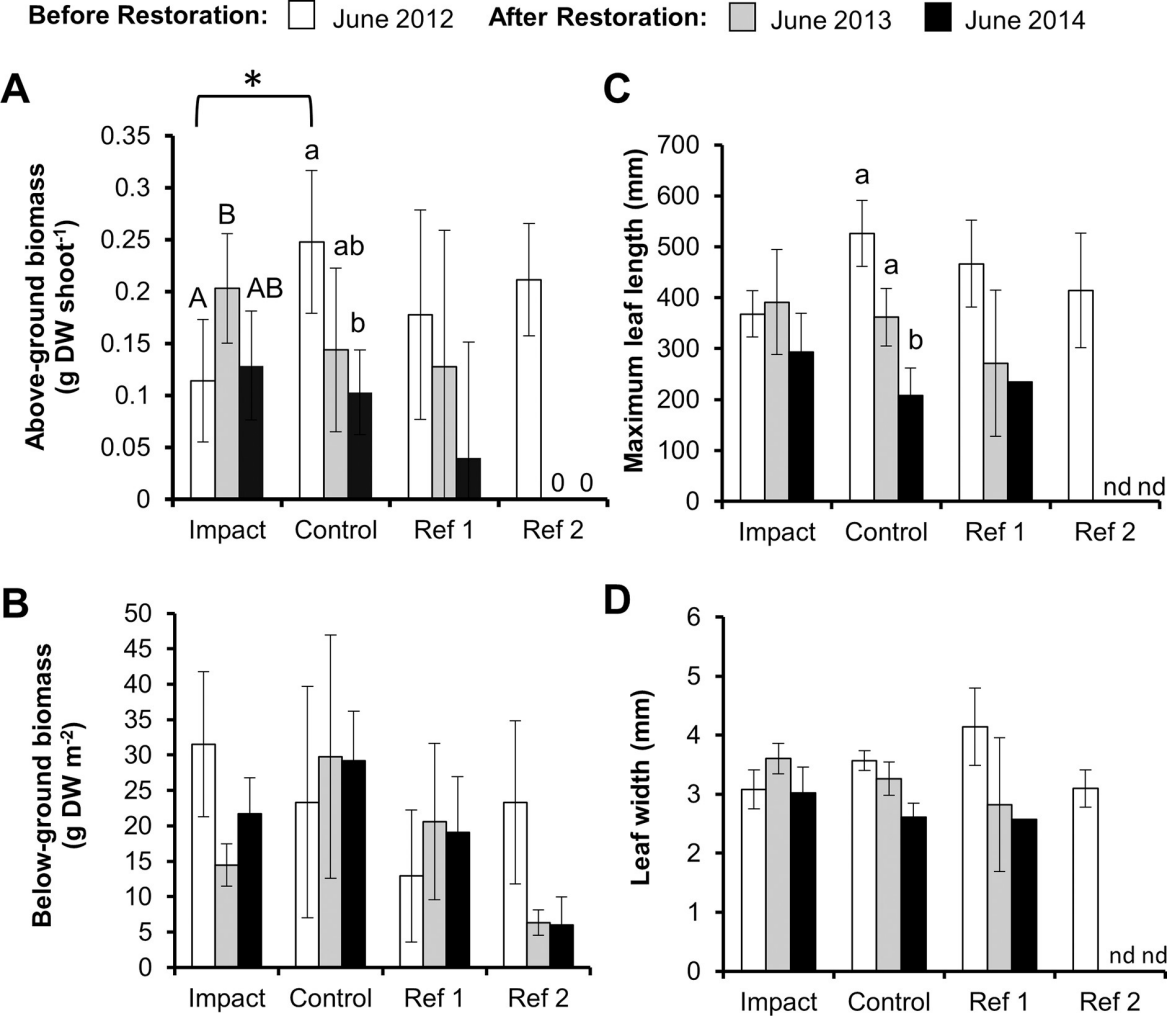
Eelgrass characteristics each year. A) Above-ground Shoot biomass; B) Below-ground biomass; C) Maximum leaf length; D) Leaf width. Values represent means with 95% confidence intervals; n = 5 samples when eelgrass shoots were present. Reference beds not included in analysis but included for comparison. Letters indicate significant differences within locations by year (uppercase—impact, lowercase—control) (S4 Table). * indicates significant differences between the control and impact location during that year (S3 Table). nd = No Data.

Collectively, eelgrass characteristics (shoot biomass, below-ground biomass, leaf length, leaf width, epiphyte load, and epiphyte percent cover) in the control and impact locations changed differently across years (PERMANOVA, Year × Location interaction: F_2,24_ = 4.39, *p* = 0.0002; Table 2, Fig 5). This was driven in part by a significant difference between the control and impact locations that existed prior to restoration (Fig 5, S3 Table). While the control and impact eelgrass showed different trajectories across years, they became indistinguishable from one another by one year following oyster bed construction (Fig 5, S3 Table). The scale-free correlation represented by the vectors in Fig 5 indicate moderately high Pearson correlations between epiphyte percent cover (0.786), leaf length (0.744), and shoot biomass (0.733) with MDS axis 1, and a 0.795 Pearson correlation between below-ground biomass and MDS axis 2. These agree with data trends and univariate statistics; for instance, leaf length and shoot biomass declined in the control location over time, and shoot biomass increased at the impact location in 2013 (Fig 6, S4 Table).

**Table 2:** Permutational ANOVA results examining the effect of oyster restoration on eelgrass response variables combined (MANOVA) and then individually (ANOVA).

		df	SS	Pseudo-F	*p* (perm) ^a^
**MANOVA**					
	Year	2	58.804	8.70	**0.0001**
	Location	1	4.398	1.30	0.25
	Year × Location	2	29.693	4.39	**0.0002**
	Residuals	24	81.105		
**ANOVA**					
**Above-ground shoot biomass**					
	Year	2	5.391	5.45	0.01
	Location	1	0.426	0.86	0.36
	Year × Location	2	11.303	11.42	**0.0004**
	Residuals	24	11.880		
**Below-ground biomass**					
	Year	2	0.844	0.44	0.65
	Location	1	1.050	1.10	0.30
	Year × Location	2	4.212	2.21	0.14
	Residuals	24	22.894		
**Maximum leaf length**					
	Year	2	13.327	15.88	**0.0002**
	Location	1	0.107	0.26	0.63
	Year × Location	2	5.492	6.54	**0.006**
	Residuals	24	10.074		
**Leaf Width**					
	Year	2	9.901	9.23	**0.002**
	Location	1	0.276	0.52	0.48
	Year × Location	2	5.950	5.55	0.01
	Residuals	24	12.872		
**Epiphyte Percent Cover**					
	Year	2	14.471	17.02	**0.0001**
	Location	1	2.044	4.81	0.04
	Year × Location	2	2.281	2.68	0.09
	Residuals	24	10.204		
**Epiphyte Load**					
	Year	2	14.870	13.54	**0.0001**
	Location	1	0.495	0.90	0.35
	Year × Location	2	0.454	0.41	0.66
	Residuals	24	13.181		

^a^ *p* values in bold are significant after Bonferroni correction *p* < 0.0083.

Above-ground shoot biomass varied between locations across years (univariate PERMANOVA, Year × Location interaction: F_2, 24_ = 11.42, *p* = 0.0004; Table 2, Fig 6A). In the impact location, shoot biomass increased one year after restoration, but returned to pre-restoration levels after two years, while shoot biomass showed a declining trend in the control location (Fig 6A, S4 Table). Shoot biomass started higher initially in the control location, but did not differ with the impact location one year after and two years after oyster bed construction (Fig 6A, S3 Table). No impact of the constructed oyster bed on below-ground biomass was detected (univariate PERMANOVA, Year × Location interaction: F_2, 24_ = 2.21, *p* = 0.14; Table 2, Fig 6B).

Leaf length differed in the magnitude of change between the locations across years (univariate PERMANOVA, Year × Location interaction: F_2, 24_ = 6.54, *p* = 0.006; Table 2, Fig 6C). Control eelgrass leaf length declined significantly by two years following oyster bed construction, whereas impact eelgrass leaf length did not show significant changes across years (Fig 6C, S4 Table). Leaf length did not significantly differ between the control and impact locations at any point during the study (Fig 6C, S3 Table). While no significant effect of the oyster bed was detected in leaf width (univariate PERMANOVA, Year × Location interaction: F_2, 24_ = 5.55, *p* = 0.01; Table 2, Fig 6D), similar trends were observed as in leaf length.

**Fig 7:**
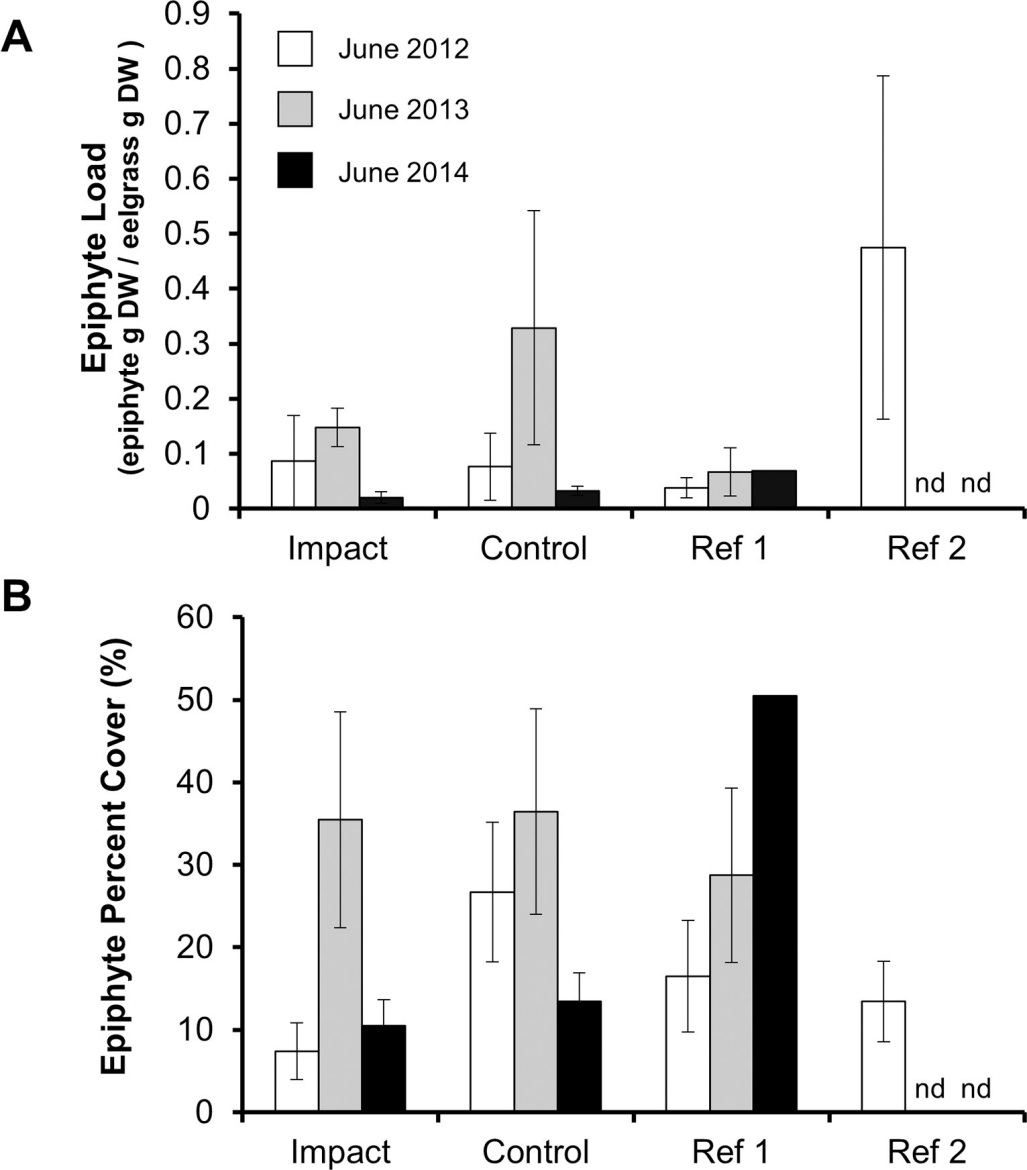
Mean epiphyte load (A) and percent cover (B) in each location each year. Values represent means with 95% confidence intervals; n = 5 samples when eelgrass shoots were present. Reference beds not included in analysis but included here for comparison. nd = No Data.

The two measures of epiphyte abundance showed no response to the constructed oyster bed. Both epiphyte percent cover and epiphyte load differed only by year (epiphyte percent cover: univariate PERMANOVA, Year: F_2,24_ = 17.02, p = 0.0001; epiphyte load: univariate PERMANOVA, Year: F_2,24_ = 13.54, p = 0.0001; Table 2; Fig 7), and not between the locations across years (epiphyte percent cover: univariate PERMANOVA, Year × Location interaction: F_2, 24_ = 2.68, *p* = 0.09; epiphyte load: univariate PERMANOVA, Year × Location interaction: F_2, 24_ = 0.41, *p* = 0.66; Table 2; Fig 7).

## Discussion

Collectively, the findings of this study suggest that construction of an Olympia oyster bed caused neither positive nor negative impacts on an adjacent eelgrass bed. Mean daily light intensity was lower at the impact location relative to the control location and one of the two reference locations; however, the difference was not greater than the naturally occurring variation among the collective reference beds and control suggesting that if light intensity on the impact eelgrass bed was impacted by oyster bed construction, it was still within the range of natural variation across all locations. Further, the constructed oyster bed produced no adverse effects on the impact eelgrass. Compared with the control and reference eelgrass beds, the impact eelgrass remained relatively stable each summer after oyster bed construction in most parameters monitored (shoot density, biomass, and leaf morphometrics).

Although no adverse impacts were observed in the impact eelgrass, different growth strategies were observed between the impact and control eelgrass over time. In the control eelgrass, a reduction in average shoot size (e.g., leaf length, shoot biomass) coupled with higher shoot density may indicate a greater proportion of younger shoots in the population produced through increased lateral branching activity. In contrast, the impact eelgrass shoot density remained stable each year, with shoot biomass increasing after one year, potentially indicating an increased allocation of energy to existing shoots rather than to the production of new shoots. This tradeoff in energy allocation has been observed before in reduced light conditions where lateral branching is decreased and the plants invest more energy into existing above-ground structures, increasing shoot size [22, 68–71].

The different growth strategies observed may be a response to the lower light conditions near the constructed oyster bed, but does not suggest a detrimental impact, as the PERMANOVA results revealed that the two locations were more similar after restoration than they were before restoration began. In addition, we did not observe declines in shoot density or biomass in the impact location that may have indicated a detrimental impact consistent with extended low light conditions. The range of densities exhibited at the impact location over time (79.2 to 287.2 shoots m^-2^) are in alignment with the previously documented range of average eelgrass densities at sites throughout Alamitos Bay (71 to 229 shoots m^-2^) [50]. By the end of the study in June 2014, the shoot density at the impact location exceeded this range (256 shoots m^-2^), giving no indication that the impact eelgrass was in poor condition. Both growth strategies have allowed each location to balance photosynthetic needs with environmental conditions and might suggest acclimatization to the addition of the oyster bed.

Similarly, we found no evidence to support the hypothesis for increased light availability on the leaf surface through oyster-mediated decreases in epiphytes. Epiphyte load and epiphyte percent cover were unaffected by the oyster restoration.

Because of limited pre-construction data, it is difficult to discern if observed variations were caused by the construction of the oyster bed or if they are natural variations typical of this system. Eelgrass shoot density and areal extent are known to fluctuate depending on a variety of factors including changes in temperature, water clarity and quality, salinity, wave and current velocities [72] and this propensity to vary is even accounted for in eelgrass management plans (e.g., California Eelgrass Mitigation Policy, City of Newport Beach Eelgrass Management Plan). Published densities of the eelgrass bed at Jack Dunster Marine Reserve (JDMR) prior to our study are limited, but varied between years and were generally lower than during our study of the site (162 shoots m^-2^ in May 1994, 52.3 shoots m^-2^ in August 2009) [73]. Coincidentally to the timing of our study, eelgrass shoot density at JDMR was also monitored between 2012 and 2014 by Tetra Tech, Inc. who similarly found shoot density to vary over time [74]. They observed shoot density decline by over 50% between June 2012 and June 2013 but increase by 40% in June 2014 [74]. This variability in shoot density within a site over time is not uncommon in the region; in nearby Newport Bay, surveys of 17 sites throughout the bay from 2003 to 2016 revealed large fluctuations in shoot density from year to year, frequently declining by half or doubling between surveys [75].

Fluctuations in shoot density in our study were present seasonally and between years in the impact and control locations but were more pronounced in the reference sites, including the complete collapse of the second reference site, complicating the detection of an effect. This variation reflects both the frequency and intensity of disturbances affecting each site but also the capacity of the populations at each site to withstand and recover from changing conditions [72]. In general, the impact eelgrass underwent fewer morphological changes after construction than the control eelgrass. These results provide some evidence that the construction of an Olympia oyster restoration project may not have a substantially positive or negative effect on an existing eelgrass bed.

The lack of a substantial impact of oyster restoration on eelgrass observed in our study may be due to several reasons. Because of the significant intake of water by the Alamitos Generating Station, the residence time of seawater in Alamitos Bay has been estimated at only one day [48]. This low water residence time may not provide the oysters enough time to clear the water column and so may limit the oysters’ potential ability to have an impact on water quality in Alamitos Bay. In addition, the potential impact of *Ostrea lurida* on water quality may be constrained by its lower filtration rate relative to other oyster species [8, 39] on which the predicted benefits of oysters on water quality have been based. However, a recent *in situ* evaluation of the filtration services provided by restored Olympia oyster beds, including an entire community of filter feeders that recruited to the beds, indicates that restored Olympia oyster habitat may have the capacity to return filtration services on par with Pacific oyster, *Crassostrea gigas*, aquaculture operations [9]; this capacity may still be modest enough to preclude any positive impact on adjacent eelgrass beds.

Additionally, several aspects of the spatial configuration of the study may have contributed to not detecting an effect. Though the control location was selected nearby the impact location to compare the effect of oyster bed presence and absence under similar conditions, the control was directly adjacent to the impact location, within the same seagrass meadow, and thus also in the vicinity of the oyster bed. Without a complete understanding of the spatial extent of potential impacts, the choice of this control location may have obscured detection of an effect if both the control and impact locations were impacted by the oyster bed restoration. This inherent limitation led to the inclusion of two additional reference beds in the study design farther from the impact and control location. Although it would not have eliminated the challenge, additional pre-restoration data would have been instructive in clarifying to what extent post-restoration trends deviated from pre-restoration trends. The lack of pre-restoration data and difficulties in selecting appropriate controls are especially common issues in assessments of environmental impacts, and the topic has been discussed in depth [e.g., 55, 56, 76].

The spatial separation between the oysters and eelgrass at different tidal heights may also dampen any potential impact. Prior studies typically assessed impacts by placing oysters directly on top of seagrass beds [e.g., 33, 36], which is not a permissible option for larger scale oyster restoration designs, nor is it representative of the primary distribution seen currently among remaining populations in southern California. Separating the two species at different tidal heights may limit the potential benefits that have been observed in prior studies, but it may also limit negative effects associated with higher densities of bivalves as well [31–33, 36]. Our study was able to achieve a higher percent cover of shell and a high Olympia oyster density [54] without a negative impact to eelgrass compared to a similar study placing oyster shell cultch within eelgrass beds [36]. At only 19% cover of shell, Archer [36] detected significant reductions in eelgrass percent cover and eelgrass shoot density. While separation may maximize densities of both species and minimize negative impacts, fewer benefits to the eelgrass appear to be returned. In contrast, Gagnon et al. [15] found through meta-analysis that plant and bivalve interactions are generally more positive when they are adjacent rather than co-located within the same area, which further emphasizes the context-dependent nature of these interactions.

Although our study did not detect any clear benefit of oyster restoration on eelgrass, there may be benefits that were not evaluated in our study. The increased habitat complexity resulting from restoring two foundation species adjacent to one another may support a more diverse community than oyster or eelgrass habitat alone. There is some mixed evidence to support this: a San Francisco Bay restoration project found greater diversity of epibenthic invertebrate assemblages when Olympia oyster and eelgrass habitats were restored at the same site (within tens of meters) but not when the two habitats were more closely interspersed (within meters) [77]. Additionally, at a restoration project in Newport Bay, the eelgrass infaunal community showed increased species richness when oysters were restored nearby [78]. While the present study only evaluated the relationship of oysters on eelgrass, evaluation of the reverse relationship may provide a more comprehensive view of ecosystem impacts [14, 15]. For example, recent research suggests that eelgrass may be able to buffer low pH conditions and ameliorate ocean acidification on local and limited timescales [79], though the impacts of this buffering on sensitive shell-forming species, like oysters, are mixed [80, 81]. Understanding the complex and multifaceted relationship between Olympia oysters and eelgrass in California should be the focus of future research.

Our study illustrates several conclusions from recent reviews on bivalve and plant/seagrass relations: in general, effects are inconsistent and not fit for generalizations [14, 15]. Though individual studies may identify clear and strong interactive effects, there are cases, like our study, where the impacts are more muted [14, 15]. This is critical to restoration design because it underscores the importance of context and case-specific information to meeting restoration goals. Existing conditions play a large role in tipping the balance of positive and negative interactions between foundation species, such as oysters and seagrass, with these interactions strongest in more stressful environments [82]. If bivalve restoration can improve poor existing conditions (e.g., eutrophication) or alleviate an existing limitation (e.g., light limitation), then the impacts to seagrass are expected to be greater (and positive). In our case, existing water clarity was already controlled by a high flushing rate due to water intakes for a nearby power plant, and thus left little room for oyster-induced improvements to water clarity. Identifying and capitalizing on conditions where the relationship between bivalves and seagrass are most positive is essential to effective co-restoration design.

Understanding the spatial scale at which interactions between bivalves and eelgrass occur is also critical to restoration design. Previous studies have found that negative impacts are reduced when the density or percent cover of oysters placed within eelgrass is low [33, 36, 83], but if the restoration goal is to achieve high densities of Olympia oysters that are self-sustaining and provide greater ecosystem services, then our study shows that spatial separation may be key. Future research is needed to determine the optimal arrangement of the two species that will maximize the benefits and minimize negative impacts on each. Considering the important ecosystem functions that both *O*. *lurida* and *Zostera marina* provide and the high potential for co-occurrence, it is crucial to find ways to protect and restore these habitats without diminishing the provisioning of these ecosystem functions.

## Supporting information

S1 Table *P* values for pairwise comparisons of shoot density between locations within each sampling time. (DOCX)

S2 Table *P* values for pairwise comparisons of shoot density between pre-restoration (June 2012) and each post-restoration sampling time within each location. (DOCX)

S3 Table *P* values from PERMANOVA pairwise comparisons between impact (I) and control (C) locations within years. (DOCX)

S4 Table *P* values from PERMANOVA pairwise comparisons between years within the impact and control locations. (DOCX)
