# Supplementary material for: Response of eelgrass (Zostera marina) to an adjacent Olympia oyster restoration project
Source: PLoS One. 2021 Oct 7;16(10):e0258119. doi: 10.1371/journal.pone.0258119 (PMC8496881; doi:10.1371/journal.pone.0258119)
Supplement: S1 Table — (DOCX) [file pone.0258119.s001.docx]

**S1 Table. *P* values for pairwise comparisons of shoot density between locations within each sampling time.**

| **Time** | **Impact - Control** | **Impact - Ref 1** | **Impact - Ref 2** |
| --- | --- | --- | --- |
| June 2012 | 0.32 | 0.004 | 0.26 |
| September 2012 | 0.26 | **0.0001** | 0.56 |
| January 2013 | 0.03 | 0.94 | **0.0002** |
| March 2013 | **0.0008** | 0.30 | **< 0.0001** |
| June 2013 | 0.06 | 0.70 | **< 0.0001** |
| September 2013 | 0.08 | 0.12 | 0.005 |
| January 2014 | **< 0.0001** | 0.33 | **< 0.0001** |
| April 2014 | 0.02 | 0.002 | **< 0.0001** |
| June 2014 | 0.02 | **< 0.0001** | **< 0.0001** |

*p* values shown in bold after Bonferroni correction (*p* < 0.00085).
