# Supplementary material for: Response of eelgrass (Zostera marina) to an adjacent Olympia oyster restoration project
Source: PLoS One. 2021 Oct 7;16(10):e0258119. doi: 10.1371/journal.pone.0258119 (PMC8496881; doi:10.1371/journal.pone.0258119)
Supplement: S2 Table — (DOCX) [file pone.0258119.s002.docx]

**S2 Table. *P* values for pairwise comparisons of shoot density between pre-restoration (June 2012) and each post-restoration sampling time within each location.**

| **Before - After** | **Impact** | **Control** | **Ref 1** | **Ref 2** |
| --- | --- | --- | --- | --- |
| June 2012 - September 2012 | 0.27 | 0.30 | 0.84 | 0.14 |
| June 2012 - January 2013 | **< 0.0001** | **< 0.0001** | 0.10 | **< 0.0001** |
| June 2012 - March 2013 | 0.87 | **0.0001** | 0.45 | **< 0.0001** |
| June 2012 - June 2013 | 0.83 | 0.06 | 0.002 | **< 0.0001** |
| June 2012 - September 2013 | **< 0.0001** | **< 0.0001** | 0.56 | **< 0.0001** |
| June 2012 - January 2014 | **< 0.0001** | 0.71 | 0.03 | **< 0.0001** |
| June 2012 - April 2014 | 0.04 | 0.82 | **0.0004** | **< 0.0001** |
| June 2012 - June 2014 | 0.97 | 0.009 | **0.0002** | **< 0.0001** |

*p* values shown in bold are significant after Bonferroni correction (*p* < 0.00085).
