# Supplementary material for: Response of eelgrass (Zostera marina) to an adjacent Olympia oyster restoration project
Source: PLoS One. 2021 Oct 7;16(10):e0258119. doi: 10.1371/journal.pone.0258119 (PMC8496881; doi:10.1371/journal.pone.0258119)
Supplement: S3 Table — (DOCX) [file pone.0258119.s003.docx]

**S3 Table. *P* values from PERMANOVA pairwise comparisons between impact (I) and control (C) locations within years.**

|  | **2012** | **2013** | **2014** |
| --- | --- | --- | --- |
|  | **I - C** | **I - C** | **I - C** |
| **All morphological metrics** | **0.009** | 0.15 | 0.06 |
| **Shoot biomass** | **0.007** | 0.12 | 0.30 |
| **Max leaf length** | 0.02 | 0.65 | 0.11 |

Pairwise comparison results for variables with significant interaction terms in the PERMANOVA (Table 2). *P* values are shown in bold when significant after Benjamini-Hochberg stepwise testing procedure was used to keep overall false discovery rate for each variable at *p* < 0.05.
