# Supplementary material for: Response of eelgrass (Zostera marina) to an adjacent Olympia oyster restoration project
Source: PLoS One. 2021 Oct 7;16(10):e0258119. doi: 10.1371/journal.pone.0258119 (PMC8496881; doi:10.1371/journal.pone.0258119)
Supplement: S4 Table — (DOCX) [file pone.0258119.s004.docx]

**S4 Table. *P* values from PERMANOVA pairwise comparisons between years within the impact and control locations.**

|  | **Impact** | | | **Control** | | |
| --- | --- | --- | --- | --- | --- | --- |
|  | **2012 - 2013** | **2012 - 2014** | **2013 - 2014** | **2012 - 2013** | **2012 - 2014** | **2013 - 2014** |
| **All morphological metrics** | **0.008** | 0.12 | **0.007** | **0.02** | **0.009** | **0.009** |
| **Shoot biomass** | **0.016** | 0.54 | 0.022 | 0.04 | **0.01** | 0.25 |
| **Max leaf length** | 0.73 | 0.13 | 0.17 | 0.02 | **0.008** | **0.008** |

Pairwise comparison results for variables with significant interaction terms in the PERMANOVA (Table 2). *P* values are shown in bold when significant after Benjamini-Hochberg stepwise testing procedure was used to keep overall false discovery rate for each variable at *p* < 0.05.
